# On Abdominal Tumors during Pregnancy

**Published:** 1852-08

**Authors:** 


### OBSTETRICS.

*On Abdominal Tumors during Pregnancy.* By Dr. Morisseau.—On two occasions, Dr. Morisseau writes, I have had an opportunity of observing the result of abdominal tumors during pregnancy. More recent facts have awakened my recollection, and fixed my attention.

A woman, aged 28, married for eight months, had become pregnant four months after marriage. For several years she had had a swelling on the right side of the abdomen. It was painless and moveable, offering no impediment to the functions of the abdomen; but it now became adherent and painful. In the seventh month, the woman died of marasmus. For a month before her death, she had had purulent alvine evacuations; but the size of the tumor appeared very slightly diminished. The tumor was found to be filled with pus, to be adherent to the mesentery, and to have an opening into the ascending colon. The fœtus was in a state of putrefaction.

A woman, aged 30, who had had two children, became pregnant for the third time. After the second pregnancy, she had what she described as a ball in her abdomen. This tumor went on growing with the product of conception; and, in the fifth month, became so painful as to render motion insupportable. After suffering extremely for four months, in a state of marasmus, she was delivered of a small, living, skeleton-like child. Both mother and infant died a few hours after. Towards the eighth month of pregnancy, I perceived a tumor of the size of the head of a fœtus at full term, seated in the right flank, and distinct from the uterine tumor. At the autopsy, the peritoneum was found to contain a large quantity of sero-purulent fluid, with albuminous flocculi floating in it. The small intestines were mottled and friable. Above the right ovary, the tumor raised the intestines, and was adherent to them by the whole of its anterior surface. It was divided into cells, which, for the most part, did not communicate, and was filled with a large quantity of very fetid greenish pus. The base was at the mesentery. The uterus appeared healthy.

Some time ago I was consulted in the case of a woman, aged 24 or 25 years, who had been married some months, to determine whether she could, without danger, become pregnant. She had for some years had an enlargement in the abdomen. The tumor was of the size of a large orange, painless, and could easily be moved from the right side, its ordinary position, to the left. I expressed my opinion that pregnancy would be attended with severe accidents; but different advice was given by another *confrere*, and the patient became pregnant. A month after conception, the tumor became painful, and larger. Towards the end of the second month the patient was obliged to remain in bed; not the least pressure could be borne; obstinate vomiting and colic set in; and, for ten days, the patient was in a most precarious state. On the seventieth day of pregnancy she expelled the fœtus. For a month she remained dangerously ill, but at length became slowly convalescent, the tumor remaining large and tender.

These facts are worthy of attention. Pregnancy necessitates increased activity of circulation in the uterus and the neighboring parts. When the uterus is developed, it impedes the flow of blood, as is proved by œdema, and varices in the lower extremities. In the abdomen, too, there is a plethoric state of the organs; hence, if a ganglion is already hypertrophied, or there be a tumor of any kind in the abdominal cavity, it will be liable to become enlarged.

Is it, then, prudent to advise marriage to a female who has a tumor in the abdomen? I have not hesitated, and would not hesitate, to reply in the negative.—*Lond. Journ. of Medicine.*

